# Pantoprazole-Induced Delirium: Review of a Case and Associated Literature

**DOI:** 10.1155/2018/1232535

**Published:** 2018-02-04

**Authors:** Anupriya Razdan, Ramaswamy Viswanathan, Alan Tusher

**Affiliations:** SUNY Downstate Medical Center, Brooklyn, NY, USA

## Abstract

**Background:**

Proton pump inhibitors (PPIs) are frequently prescribed antiulcer agents in hospitals and are shown to be safer than H-2 blockers. We present a case report of PPI-induced delirium, regarding which not much has been written in the literature.

**Case Report:**

We present a case of a 93-year-old woman with no known past psychiatric history, who was hospitalized for syncope workup and who developed delirium after a double dose of pantoprazole.

**Discussion:**

Very few reports of PPI-induced delirium exist in the literature. In this case report, we attempt to highlight the mechanism of PPI induced delirium which in our case was most likely due to the primary effects of PPI and drug-drug interactions. Given the paucity of literature on this topic, we encourage further research into relationship between PPI and delirium and urge caution while using PPIs in geriatric population.

## 1. Case Report Introduction

Proton pump inhibitors (PPIs) are frequently prescribed antiulcer agents in the hospital. One of the most commonly used PPIs is pantoprazole. PPIs are generally considered safer and more efficacious than H2 blockers as antiulcer agents [1, 2]. A study showed reduced incidence of antiulcer drug associated delirium when medications were switched from H-2 blockers to proton pump inhibitors [3]. While there is plenty of literature supporting the use of pantoprazole as an effective antiulcer agent, only one case of delirium associated with PPIs, omeprazole, has been reported [4]. There are three reports of central nervous system dysregulation reported on omeprazole [5]. There is no reported case of pantoprazole associated delirium in the literature. In our report, we present a case of a 93-year-old woman with no known past psychiatric history and medical history of hypertension and arthritis, who developed symptoms of delirium including visual hallucinations while on pantoprazole.

## 2. Method

literature search was done using keywords like pantoprazole, delirium, proton pump inhibitors, and the central nervous system using search engines like PubMed and PsycINFO.

## 3. Case Report

Ms. X was a 93-year-old woman with no known past psychiatric history and medical history of essential hypertension and arthritis. She was admitted to our hospital's medical unit for syncope workup following a fall due to syncope.

On day 1 of her admission, the patient was alert and oriented to time, place, person, and situation.

Laboratory workup done on admission was within normal limits except for low vitamin D level of 25.68.

Chest X-ray done was normal. CT head showed evidence of atrophy of brain and microvascular disease.

MRI of the cervical spine showed moderate spondylosis and mild subluxation on a degenerative basis. Moderate central canal stenosis and cord compression C2-T1 with edema and cervical cord compression were noted at C3 and C6 level.

The patient was evaluated by a neurosurgeon who recommended 6–8 weeks of cervical collar and treatment with dexamethasone along with proton pump inhibitor (PPI) for ulcer prophylaxis. No additional workup was recommended.

The patient was started on her home medications, which consisted off amlodipine 10 mg daily and oxybutynin 5 mg daily. She was started on dexamethasone 2 mg IV q 12 hrs.

On day 2 of admission, the patient received two doses of pantoprazole over a period of 5 hours to a total dose of 80 mg on day 2.

On day 3, the patient was observed to be confused, with acute changes noted in her behavior by staff and family. The patient reported auditory and visual hallucinations and was observed to be internally preoccupied. She was observed to be resisting evaluation and refusing her medications. Diurnal variation in her symptoms was noted with worsening of symptoms during evening and night. The patient did not receive pantoprazole that day as she refused her medications. Dexamethasone was stopped in the evening by the primary team as it was believed to be causing the hallucinations.

On day 4, the patient continued to be confused and alert but not oriented to time, place, person, or situation. She was aggressive with staff at times and was noted to be talking to herself, internally preoccupied, and refusing her medications. Psychiatry consult team was called to evaluate the patient for these sudden behavioral changes. During the interview with the psychiatrist, she was alert but not oriented to time, place, or person. She was seen to be talking to an imaginary person in the room who was “sitting on the roof,” asking him not to “pull” her hair, and told the writer that she has met her 8 years back in New York (not true). The family denied the patient having any past psychiatric history or history of hallucinations. It was noted that patient had been living alone and had a fair level of premorbid functioning. It was recommended that pantoprazole be stopped and switched to H-2 blockers.

On day 5, pantoprazole was stopped and the patient was started on ranitidine. She continued to be confused at times but no hallucinations were reported by the nurses. The patient showed an improvement in her behavioral symptoms. Dexamethasone was restarted this day with decreased dose of 2 mg IV daily. Due to a gradual improvement in symptoms, the need for an EEG was not felt necessary.

On day 6, the patient was alert and oriented to time, place, person, and situation. As per family and primary team, she appeared to be at her baseline functioning, behaviorally.

Dexamethasone along with her other medications was continued throughout the hospital course. The patient was in the hospital for 16 days for treatment with dexamethasone with no relapse in her delirious symptoms.

## 4. Discussion

Delirium is defined as a disturbance in cognition and attention, developing over a short period of an alternating course. The exact pathophysiology of delirium is not fully understood. However, few hypothesis has been proposed to explain the neurobiology behind it. As per the neurotransmitter hypothesis, abnormalities in various neurotransmitters like decreased cholinergic function, an excess of dopamine, norepinephrine and glutamate release, and both decrease and increase in serotonergic and GABA may be associated with cerebral dysfunction and consequently decreased oxidative metabolism leading to the symptoms of delirium. Another theory supports the role of inflammation and release of cytokines in the causation of delirium due to increased permeability of membranes and influx of cytokines causing disruption of neurotransmitter system. Increased cortisol release during stress may also act on the serotonergic (5-HT1A) receptor in the hippocampus and contribute to the symptoms of delirium [6].

The elderly are especially sensitive for the development of delirious symptoms while in the hospital due to multiple comorbid conditions and risk factors. Literature shows that delirium itself is known to be an independent predictor of adverse outcomes in elderly hospitalized patients.

Proton pump inhibitors inhibit the final step in gastric acid production by irreversibly binding with gastric H+/K+-ATPase (proton pump) and preventing acid release [6].

The exact mechanism responsible for delirium in our patient is not clear but could have been the result of multiple factors acting in concert. Drug-drug interactions (DDI) were less likely as no significant DDI was noted amongst the patient's medications [7].

Pantoprazole is a relatively safe drug but package insert for the drug mentions reports of rare psychiatric side effects like hallucination, confusion, insomnia, and somnolence in the postmarketing period [8].

Dexamethasone is a short-acting and potent steroid commonly associated with psychiatric side effects ranging from euphoria, insomnia, mood swings, personality changes, and severe depression to frank psychotic manifestations [9].

In our case, Ms. X did have some factors in her history that made her more vulnerable to developing delirium like increased age, medical comorbidity, use of high potency steroids, and multiple medications uses. Her timeline for onset of her delirious symptoms and concomitant use of a double dose of pantoprazole and starting dexamethasone made it difficult to determine the cause of her symptoms. Pantoprazole and dexamethasone both were stopped and her symptoms resolved. However, once her symptoms resolved, dexamethasone was restarted with no rebound of her delirious symptoms. This made us think that pantoprazole was the most likely causative agent of her delirium and not dexamethasone. However, dexamethasone cannot be fully ruled out as a causative agent.

## 5. Conclusion

Delirium is an important medical condition that is easy to miss or overlook in the medical setting. However, it is important to diagnose it early and address the factors causing it and ensure that the patient and staff are safe. Educating the primary team and the family about the condition and its presentation is important to avoid any biases amongst caregivers and to ensure that the patient gets the best care possible. Patient and family need to be reassured that the behavioral manifestations of delirium will resolve with the condition. Due to the limited literature on this subject, we encourage clinicians to report any such cases found in their practice to build a database for such rare but treatable side effects of the medication.
